# Individual, clinical and system factors associated with the place of death: A linked national database study

**DOI:** 10.1371/journal.pone.0215566

**Published:** 2019-04-18

**Authors:** Woan Shin Tan, Ram Bajpai, Chan Kee Low, Andy Hau Yan Ho, Huei Yaw Wu, Josip Car

**Affiliations:** 1 Centre for Population Health Sciences, Lee Kong Chian School of Medicine, Nanyang Technological University, Singapore, Singapore; 2 NTU Institute for Health Technologies, Interdisciplinary Graduate School, Nanyang Technological University, Singapore, Singapore; 3 Health Services and Outcomes Research Department, National Healthcare Group, Singapore, Singapore; 4 Economics Programme, School of Social Sciences, Nanyang Technological University, Singapore, Singapore; 5 Psychology Programme, School of Social Sciences, Nanyang Technological University, Singapore, Singapore; 6 Palliative Care Centre for Excellence in Research and Education, Singapore, Singapore; 7 Department of Palliative Medicine, Tan Tock Seng Hospital, Singapore, Singapore; 8 Global eHealth Unit, Department of Primary Care and Public Health, School of Public Health, Imperial College London, London, United Kingdom; Institute of Mental Health, SINGAPORE

## Abstract

**Background:**

Many middle- and high-income countries face the challenge of meeting preferences for home deaths. A better understanding of associated factors could support the design and implementation of policies and practices to enable dying at home. This study aims to identify factors associated with the place of death in Singapore, a country with a strong sense of filial piety.

**Settings/participants:**

A retrospective cohort of 62,951 individuals (≥21 years old) who had died from chronic diseases in Singapore between 2012–2015 was obtained. Home death was defined as a death that occurred in a private residence whereas non-home deaths occurred in hospitals, nursing homes, hospices and other locations. Data were obtained by extracting and linking data from five different databases. Hierarchical multivariable logistic regression models were used to examine the effects of individual, clinical and system factors sequentially.

**Results:**

Twenty-eight percent of deaths occurred at home. Factors associated with home death included being 85 years old or older (OR 4.45, 95% CI 3.55–5.59), being female (OR 1.21, 95% CI 1.16–1.25), and belonging to Malay ethnicity (OR 1.91, 95% CI 1.82–2.01). Compared to malignant neoplasm, deaths as a result of diabetes mellitus (OR 1.93, 95% CI 1.69–2.20), and cerebrovascular diseases (OR 1.28, 95% CI 1.19–1.36) were also associated with a higher likelihood of home death. Independently, receiving home palliative care (OR 3.45, 95% CI 3.26–3.66) and having a documented home death preference (OR 5.08, 95% CI 3.96–6.51) raised the odds of home deaths but being admitted to acute hospitals near the end-of-life was associated with lower odds (OR 0.92, 95% CI 0.90–0.94).

**Conclusion:**

Aside from cultural and clinical factors, system-based factors including access to home palliative care and discussion and documentation of preferences were found to influence the likelihood of home deaths. Increasing home palliative care capacity and promoting advance care planning could facilitate home deaths if this is the desired option of patients.

## Introduction

Many middle- and high-income countries are currently facing the challenge of providing quality end-of-life care, and meeting patients’ preferences for dying at home. In a systematic review of 210 studies from 34 countries, 75% revealed that most people prefer to die at home. Home death preferences were found to be relatively stable, as only one in five studies reported changes as the patient's disease progressed [1]. Despite a consistent preference for dying at home across these advanced societies, most decedents died in hospitals. Similarly, in Singapore, the rate of home death has been falling, despite a majority of Singaporeans (77%) indicating home death preferences [2]. In fact, 49% of Singaporean decedents died at home in 1965, as compared to 33% in 1990, and 25% in 2015 [3].

Singapore has a multiethnic population of 5.5 million comprising Chinese (74%), Malay (13%), Indian (9%), and individuals belonging to other ethnic origins (3%) [4]. Like other high-income countries, Singapore is experiencing rapid population ageing. Based on government estimates, 19% of the resident population will be 65 years or older in 2030 [5]. With individuals of Chinese ethnicity forming the majority of the population, there is strong subscription to the principle of filial piety, which is premised upon the belief that children must obey and care for their parents [6]. Through socialisation, the moral obligation to care for one’s elderly parents, is upheld also by other ethnic groups [7]. With the passing of the “Maintenance of Parents Act” in 1995, this obligation also became a legal one, as parents aged 60 years and over, can seek maintenance from their children [7]. With the strong societal and policy emphasis on the family as the main source of support, most aged care occurs at home [8] rather than in aged care facilities.

The preference to die at home is fundamentally driven by the wish to be surrounded by loved ones in a familiar setting when death approaches [9]. Being at home also allows one to maintain autonomy and control over one’s daily life [10], an important aspect of dignity that could be compromised in institutional settings where individuals’ livelihoods are determined by the healthcare system [11, 12]. Many patients experience a gradual loss of dignity through institutional dependency, and this could further distress them and lower their quality of life as dying approaches [13]. At the same time, individuals whose family members received adequate formal or informal end-of-life support, that enabled death at home, reported less intense grieving, both at the time of death and in post-bereavement, as compared to relatives of those who died in the hospital [14].

The place of death is the result of a complex interplay between individual characteristics (socio-demographic), nature of the illness, palliative care policies, and societal perceptions of the desired place of death [15]. Governments across higher-income countries have enacted healthcare coverage and financing policies to support care and death at home. In the US, the enactment of policies, such as the Medicare hospice benefit in the 1980s and the Patient Self-Determination Act in 1990 may influenced the share of deaths at home. The percentage of home deaths rose from 17% in 1980 [16] to 31% in 2017 [17]. Similar policy efforts [18] to reverse the tide of hospital deaths in the UK was also associated with an increase in the share of home deaths [19]. In Singapore, the government has adopted a multi-pronged approach to raise the quality of palliative care services, and to expand the capacity and affordability of home-based palliative care services. In 2011, Singapore implemented a systematic Advance Care Planning (ACP) program nationally, which aimed to promote and support discussions between individuals, their families and healthcare providers about future end-of-life care plans [20].

Many studies have examined factors that influenced dying at home. Gomes & Higginson [21] reviewed evidence from 58 studies. Among cancer patients, functional impairment, preference to die at home, having access to home care, high home care intensity, residing with relatives, and having support from the extended family were found to increase the likelihood of dying at home. A later study by Costa et al., that extended the review to non-cancer populations [22], additionally highlighted the importance of policy-amendable factors, such as the provision of home palliative care by a multidisciplinary team, provision of palliative care services in nursing homes, and timely referrals to palliative care services. While three studies have been conducted in Singapore on the correlates of home death [23–25], the factors explored were mostly patient-related and non-modifiable. Although Poulose et al. found that earlier referral to an inpatient palliative care service was positively associated with dying at home, the sample was relatively small and based at a single site [25].

In this study, besides individual and clinical factors, we aimed to examine the influence of having a known home death preference, home palliative care use, and acute hospital use close to the end-of-life. Using data from different national administrative and clinical databases, this population-based study sought to identify the factors influencing deaths at home for individuals who had died between 2012 and 2015.

## Methods

This is a retrospective cohort study, which included all adults (≥21 years old) who had died of malignant neoplasm, diabetes mellitus, heart and hypertensive disease, cerebrovascular disease, and lung and respiratory diseases between January 2012 and December 2015 in Singapore. We have excluded deaths due to infectious and parasitic diseases, accidents and violence, and other causes. This study was reported using the Strengthening the Reporting of Observational Studies in Epidemiology (STROBE) guidelines [26].

### Sources of data

To assemble the required data variables, five different databases were linked (Table 1). All data were linked and anonymized using a project unique identifying number, which was generated based on the deceased’s National Registration Identity Card number. The final data set contained information on each decedent’s date of birth, date of death, sex, ethnic group and place of death; preferred place of death as documented in an ACP; information on each public hospital admission episode, including the date of admission and discharge, as well as diagnoses; and also, intermediate and long-term care service utilization, including home palliative care, inpatient and nursing home admissions.

**Table 1:** Data sources that were linked.

Data sources	Variables
MOH registry of death database	Age, sex, ethnic group, date of birth, date of death, cause of death, place of death
MOH case mix and subvention database	Date of hospital admission and discharge; ICD-9-CM and ICD-10-CM diagnosis codes
MOH intermediate and long-term care information systems	Nursing home admissions; hospice admissions; home palliative care use
National ACP IT system	Patient preferences for home death
TTSH hospital ACP database	Patient preferences for home death

ACP: Advance Care Planning; ICD-9-CM: International Classification of Diseases, 9th version with clinical modification; ICD-10-CM: International Classification of Diseases, 10th version with clinical modification; IT: Information Technology

### Outcome variable

In this study, we have defined the dependent variable “home death” as a death that had occurred at a private residential address. Deaths occurring at other sites were grouped as “non-home death”. These include locations such as private and public hospitals, nursing homes, residential aged care facilities for the destitute, and inpatient hospices. In Singapore, 25% of all deaths occurred in private residential homes, 62% of all deaths occurred in the hospital, 6% in nursing homes and aged care facilities, 4% in inpatient hospices, and 3% in other locations [3].

Other studies [22] have increasingly considered nursing home deaths to be similar to home deaths as these residential facilities eventually become their homes. However, in the Singaporean context, both nursing homes and charitable institutions are typically operated by charitable organizations where the living quarters are organized in dormitory style and the residents may have little privacy and have ‘little more than a bed, cabinet and toothbrush to call their own’ [27].

In reflecting that death in an acute hospital, nursing homes and residential aged care facilities may be less desirable than home, we have opted to group non-home deaths together as a shift in place of death towards home will be desirable in the current long-term care landscape.

### Independent variables

In this study, variables found to be significant in the literature [21–24] were extracted from the above databases. The independent variables were classified according to the theoretical framework established by Gomes and Higginson on factors influencing the variations in the place of death [21]. Specifically, the model comprised three constructs encapsulating (i) individual factors, which pertain to characteristics that defines the person; (ii) factors related to illness referred to health status changes experienced by the individual; and (iii) system factors comprised contextual elements including healthcare input received by the patients.

Individual factors included age group, gender, and ethnic group (Chinese, Malay, Indian, Others). In the multivariable analysis, we have grouped the ethnic groups of Chinese, Indians and Others together as the share of home deaths did not vary significantly between the Chinese and Indians. The category of “Others” had low numbers of home deaths and was also grouped together to prevent over estimation in the model.

Clinical factors included the primary cause of death, and the extent of comorbid burden. We have coded the primary causes of death, using the International Classification of Diseases’ 9^th^ and 10^th^ codes with clinical modification (ICD9CM, ICD10CM), according to the official local classification [3]. Comorbid burden was computed using the Charlson Comorbidity Index (CCI) [28, 29]. Looking back three years from the date of death, principal and secondary diagnosis codes for all hospital admissions incurred by the individual were identified to compute the CCI. The CHARLSON command in Stata was used [30].

System factors included previous hospital admissions, home palliative care use, and “known home death preferences” as this variable reflected the availability of ACP to patients to document their preferences. Home-based palliative care was defined and computed as a variable that measured access to a home palliative care service, at least 30 days prior to death. We also included a variable that indicated a documented preference to die at home in an ACP. Access was measured in terms of utilization in this study, which in turn is dependent on referral to home palliative care, meeting the admission criteria of the service, and having the family accepting the services. In addition, the number of admissions to hospital in the period 90–30 days before death was included as a factor in the model as contact with hospitals.

Individual data (date of birth, date of death, sex, ethnic group) were obtained from the Registry of Births & Deaths. As all deaths in Singapore must be registered within three days of occurrence according to the Registration of Births and Deaths Act, this set of data is relatively complete except for 68 cases of missing dates of birth. Similarly, as a doctor or authorised medical practitioner must issue the certificate of cause of death as part of death registration process, there were no cases with missing causes of death.

For clinical data pertaining to the computation of the Charlson Comorbid Index, and the specification of system-related data such as the incidence and number of hospital admissions home palliative care use and recorded home death preference, as with most retrospective database studies, we were unable to differentiate missing data from a real absence of a particular diagnosis or health service utilisation. Therefore, we made a simplifying assumption that the lack of recorded information referred to an absence of the diagnosis or service use or home death preference.

### Statistical analysis

Continuous variables were summarized using means (± SD) and categorical variables using frequencies and percentages. We removed 68 cases from the analysis as their date of birth was not available in electronic health records. We first carried out bivariate analyses (chi-square for categorical and ‘t’ test for continuous variables) to examine the relationship between home death and each of the variables. Hierarchical multivariable logistic regression models were then used to examine the relationship between a dichotomous dependent variable (home death or not), and independent variables measuring individual (age, sex, ethnic group) in Model I; the added impact of clinical factors (comorbid burden, cause of death) and system factors (home death preference, home palliative care use, acute hospital admission in 30–90 days before death) in Models II and III respectively.

The p-value statistic is not useful in this study as it concerns population-level data so any observed difference is an actual difference. Thus, for the bivariate analysis, the differences in proportions were compared. For the logistic regression model, we have chosen to report the OR for each of the independent variables, as well as the corresponding 95% confidence intervals (CIs). The CIs provided an indication of the direction and magnitude of the effect [31].

Additionally, with a large data set, we have opted not to rely on the traditional measures of the goodness-of-fit model, such as the Hosmer-Lemeshow (HL), because assessments by deciles have been shown to be unreliable for sample sizes exceeding 25,000. For example, the HL test statistic becomes very sensitive to small departures from the expected distribution [32]. Therefore, to assess the fit of the overall model, we have used the area under the curve (AUC) [33] to determine whether the fitted model predicted home death adequately. An AUC of 0.5 indicates that the model performed no better than chance. We also checked the Akaike Information Criterion (AIC) and (BIC) for each fitted model to judge best predictive model for home death [34]. All statistical analyses were carried out using Stata version 12 [35].

Ethics approval was obtained from the institutional review board of Nanyang Technological University and Domain Specific Review Board of the National Healthcare Group, Singapore.

## Results

A total of 76,927 deaths occurred in Singapore between 2012 and 2015. For our analysis, we have excluded 1,209 as they were aged 21 years old or less at the point of death, 68 were excluded due to missing data. Of the 75,650 complete cases of adult deaths, 12,699 individuals were excluded from the analysis as they died from infectious and parasitic diseases, accidents and violence, and other causes. Nineteen percent of these excluded deaths occurred at home whereas the place of death due to chronic diseases are reflected in Table 2. The final analytical sample included 62,951 decedents. In this sample, 28% of these decedents died at home.

**Table 2:** Profile of decedents and bivariate analysis of place of death (n = 62,951).

Variables	Total (column %)	Home deaths (column %)	Non-home deaths (column %)	p-value*
**Individual factors**				
Age (years)				<0.001
21–34	620 (1.0)	95 (15.3)	525 (84.7)	
35–44	1,480 (2.4)	302 (20.4)	1,178 (79.6)	
45–54	4,504 (7.1)	956 (21.2)	3,548 (78.8)	
55–64	9,964 (15.8)	2,388 (24.0)	7,576 (76.0)	
65–74	13,341 (21.2)	3,487 (26.1)	9,854 (73.9)	
75–84	17,723 (28.2)	5,318 (30.0)	12,405 (70.0)	
≥85	15,319 (24.3)	5,388 (35.2)	9.931 (64.8)	
Mean age ± SD (years)	74.0 ± 14.3	76.4 ± 13.7	73.0 ± 14.5	<0.001
Sex				<0.001
Female	28,728 (45.6)	9,243 (32.2)	19,485 (67.8)	
Male	34,223 (54.4)	8,691 (25.4)	25,532 (74.6)	
Ethnic group				<0.001
Chinese	48,350 (76.8)	13,451 (27.8)	34,899 (72.2)	
Malay	8,787 (14.0)	3,249 (37.0)	5,538 (63.0)	
Indian	4,431 (7.0)	983 (22.2)	3,448 (77.8)	
Others	1,383 (2.2)	251 (18.2)	1,132 (81.8)	
**Clinical factors**				
Cause of death				<0.001
Malignant neoplasm	22,813 (36.2)	7,434 (32.6)	15,379 (67.4)	
Heart & hypertensive disease	16,400 (26.1)	3,390 (20.7)	13,010 (79.3)	
Lung & respiratory disease	16,338 (25.9)	4,047 (24.8)	12,291 (75.2)	
Cerebrovascular disease	6,360 (10.1)	2,599 (40.9)	3,761 (59.1)	
Diabetes mellitus	1,040 (1.7)	464 (44.6)	576 (55.4)	
Charlson comorbid index				<0.001
≤1	18,974 (30.1)	6,498 (34.3)	12,476 (65.7)	
= 2	5,481 (8.7)	1,592 (29.1)	3,889 (70.1)	
≥3	38,496 (61.2)	9,844 (25.6)	28,652 (74.4)	
**System factors**				
Hospitalisations in 30–90 days before death	31,387 (49.9)	4,851 (15.2)	26,536 (84.8)	<0.001
Recorded home death preference	622 (1.0)	322 (51.8)	300 (48.2)	<0.001
Home palliative care use	7,112 (11.3)	3,556 (50.0)	3,556 (50.0)	<0.001

CCI: Charlson Comorbidity Index

*chi-square test was used to assess the differences between groups

### Bivariate results

From Table 2, compared to those who did not die at home, there was a higher proportion of individuals who were aged older than 75 years and who were female among those who did die at home. Relative to other races, a Malay death is more likely to be at home. Comparing amongst different causes of death, a higher proportion of those who died at home died from malignant neoplasms and cerebrovascular disease. A relatively higher proportion of those who died at home had lower comorbid burden, and were not admitted an acute hospital near the end-of-life. Utilization of home palliative care, and having a documented preference for dying at home were found to be also more likely among the home death group. In this sample, 1,611 individuals stated their preferences about place of death, of which 39% stated home as their preference.

### Logistic regression results

The results of the hierarchical multivariable logistic regression models are presented in Table 3. From Models I and II, older age, being female and being Malay were positively associated with higher odds of dying at home. With the addition of clinical factors and system factors, the directionality of the individual factors remained unchanged. However, the inclusion of system factors did shift the directionality of the relationship between cerebrovascular diseases and home deaths relative to malignant neoplasms.

**Table 3:** Multivariable hierarchical logistic regression analysis of factors associated with home death* (n = 62,951).

Variables	Model I		Model II		Model III	
	OR	95% C.I.	OR	95% C.I.	OR	95% C.I.
**Individual factors**						
Age (years)						
[21–34]	1.00	-	1.00	-	1.00	-
35–44	1.44	1.21–1.71	1.46	1.13–1.89	1.44	1.11–1.87
45–54	1.55	1.33–1.80	1.63	1.29–2.07	1.55	1.22–1.96
55–64	1.85	1.60–2.13	2.12	1.69–2.67	2.04	1.62–2.57
65–74	2.13	1.85–2.46	2.59	2.07–3.25	2.48	1.98–3.12
75–84	2.55	2.22–2.94	3.41	2.72–4.28	3.28	2.61–4.12
≥85	3.22	2.79–3.71	4.59	3.66–5.76	4.45	3.55–5.59
Sex						
[Male]	1.00	-	1.00	-	1.00	-
Female	1.28	1.23–1.31	1.22	1.18–1.27	1.21	1.16–1.25
Ethnic group						
[Non-Malay]	1.00	-	1.00	-	1.00	-
Malay	1.65	1.57–1.72	1.91	1.82–2.01	1.91	1.82–2.01
**Clinical factors**						
Cause of death						
[Malignant neoplasm]			1.00	-	1.00	-
Diabetes mellitus			1.42	1.25–1.62	1.93	1.69–2.20
Heart & hypertensive disease			0.36	0.34–0.38	0.51	0.48–0.54
Cerebrovascular disease			0.90	0.85–0.96	1.28	1.19–1.36
Lung & respiratory disease			0.39	0.37–0.41	0.54	0.51–0.57
Charlson comorbid index						
[≤1]			1.00	-	1.00	-
= 2			0.63	0.59–0.68	0.62	0.57–0.66
≥3			0.45	0.43–0.47	0.42	0.40–0.44
**System factors**						
Number of hospitalisations in 30–90 days before death					0.92	0.90–0.94
Recorded home death preference						
[No]					1.00	-
Yes					5.08	3.96–6.51
Home palliative care use						
[No]					1.00	-
Yes					3.45	3.26–3.66

*adjusted for the year that the death occurred; [.]: Reference group; OR: odds ratio; CCI: Charlson Comorbidity Index; CI: confidence interval

Based on the full model represented by Model III, compared against the reference group (21–34 years old), the 95% confidence intervals of the odds of a home death for other older age groups were greater than 1. The odds of experiencing a home death also increased as age increased. Compared to males, the odds of a home death were higher for females. The odds of a home death among Malays was 1.91 times the odds for non-Malays. Compared to individuals who died of malignant neoplasm, those who died from diabetes mellitus, and cerebrovascular disease had a higher OR of a home death; whereas individuals who died due to heart or lung diseases were less likely to experience a home death. The odds of a home death for individuals with a CCI score of three or higher was 0.42-times the odds of those with scores that were 1 or below.

An acute hospital admission in the 30–90 days prior to death was associated with a lowered likelihood of a home death. On the other hand, home palliative care recipients, as well as individuals who had documented a preference for dying at home in an ACP document, faced increased odds of dying at home. The area under the estimated ROC curve (AUC) was 0.695 (95% CI 0.690–0.699) for model III (0.593 for model I; and 0.662 for model II respectively) and decreasing AIC and BIC values, indicating the model had fair discriminatory power and better predictive ability.

## Discussion

This study found that among adult decedents in Singapore, the likelihood of dying at home increased with certain individual and clinical factors, including age, being female, of Malay ethnicity, and lowered comorbid disease burden. Compared to individuals who died from malignant neoplasm, those who died from diabetes mellitus and cerebrovascular diseases were more likely to have died at home. Completion of advance statements and home palliative care, also contributed to a higher possibility of home death but admissions to acute hospitals near the end-of-life reduced this likelihood.

Other Asian studies similarly found that older individuals tended to die at home [23, 24, 36–38] but our results are different from trends observed in countries such as Scotland [39] where the percentage of deaths occurring at home decreased with age. Cultural differences in caregiving and its influence on the role of residential care homes as a place of care for the elderly are likely to be important here. In Singapore, residential care homes or nursing homes cater only for 2% of the total elderly population in Singapore [40] because the Singaporean culture and healthcare policies are largely geared to support the ageing-in-place and caring for one’s family members in a home environment [41, 42].

The last place of residence where the patient was cared for, is usually also the place of death. In terms of gender influence, our results reiterated the findings from two other Singaporean studies [23, 24] can be attributed to the higher propensity of hospitalization for males in Singapore [43]. Compared to a diagnosis of cancer, cardiovascular and respiratory diseases had the opposite impact [22] on home deaths. One plausible explanation is that patients who died of cardiovascular accidents are more likely to present with unexpected acute deterioration or collapse as a terminal event [44]. Hospitalization is also required for pneumonia, which accounted for 87% of deaths due to lung and respiratory diseases. Our results also showed that end-of-life care of individuals with multiple comorbid disease conditions tend to be complex, with frequent admissions to acute care facilities, leading to a lower likelihood of dying at home [37].

Ethnic minorities, with lower access to primary care [45] home care [21], and a lower rate of advance directive completion [45], are often less likely to die at home. However, our study found that individuals of Malay ethnicity, which formed 15% of the Singaporean population, had a higher likelihood of home deaths. A complex interplay of socioeconomic position and cultural factors could have contributed to more care being delivered at home, which may have influenced the place of death. First, Malay families have a bigger family size of 3.9 persons per household compared to the national average of 3.4, and close to 70% of older Malays resided with their children compared with 60% observed for Chinese and Indians. Second, although 50% of the Singaporean working population earned more than USD 2,923 per month (SGD 4,000 at the rate of USD 1 = SGD 0.73), only 32% of Malay individuals earned an income above this level. Since co-payments are a key feature of the Singaporean healthcare financing system [46], affordability of formal healthcare could be a challenge. Third, individuals of Chinese and Indian ethnicity also seemed to utilize long-term residential care more [47, 48] than Malays, which could have contributed to our findings as well.

Unsurprisingly, system factors amenable through policy exert an influence on the place of death. Singapore has made significant progress towards ensuring a good quality of end-of-life care. Having previously stated a home death preference in an ACP document was also strongly associated with a higher likelihood of dying at home. Our results indicated that 51% of decedents with a stated home preference died at home, which doubles the national figure. However, the percentage of decedents who have a documented ACP preference to die at home is low because few individuals have completed this end-of-life discussion and finalized their preferences [49]. Previous studies [21, 22] have reported similar results. The statement of a preference acts as a form of goal-setting, and when effectively communicated to family members and healthcare providers, actions could be taken to honor these preferences. Additionally, our study identified access to home palliative care to be associated with dying at home. This concurred with the findings of a Cochrane review, which found home palliative care to more than double the likelihood of dying at home, and that to enable one additional death at home, five additional individuals need to receive home palliative care [50].

### Strengths and limitations

A key strength of our study lies in linking population-based information about the place of death with national data based on the ACP, and acute and long-term care utilisation. Gomes and Higginson [21], and Costa et al. [51] have earlier illustrated the importance of social support measures. However, we were unable to incorporate these variables into our model. Future studies should strive to include such variables, since changes to household structures and caregiving norms could significantly influence the place of death.

A key limitation of this study is the lack of data that measured whether the propensity to receive palliative home care is linked to a preference for home death and the completion of an ACP conversation. Further research would be required to substantiate our preliminary observations that ACP was associated with a higher likelihood of home deaths.

By exploring the factors associated with home deaths, one might infer that home deaths are assumed to be “more ideal”, but we do recognize that individuals may also prefer to die in other settings. Whilst recognizing that place of death preferences vary, examining the correlates of home death and the impact of policies that enable it remains important, due to the attention paid in policy-making to fulfill preferences to die in the comfort of one’s home [52]. Future studies should further examine the multiple factors influencing unmet preferences for home deaths, and pay attention to ensuring a high quality of death in an institution.

### Implications for practice and policy

Singapore is one of the first Asian countries to have implemented ACP at the national level, with the government aiming to reach out to and initiate ACP conversations with 100,000 Singaporeans [53], expanding the focus of the programme to beyond those diagnosed with advanced illnesses. This may significantly impact the proportion of home deaths in the future, if it is indeed preferred by the population. Beyond Singapore, ACP, as a platform for patients to communicate their end-of-life care preferences with caregivers and healthcare professionals, could support the meeting of home death preferences in other Asian countries. In tandem, our results support the Singapore government’s efforts to grow the capacity of home palliative care providers to meet preferences for home death. The number of home palliative care sites increased from 3,800 in 2011 to 5,500 in 2016, with a target of reaching 6,000 by 2020 [53]. Greater focus could be placed on developing services for non-cancer populations, such as heart disease, to reduce any differentials currently observed.

## Conclusion

Our study illuminated the importance of home-based palliative care and ACP, as well as cultural and illness-specific factors in contributing to the eventual place of death. Our results imply that such policy initiatives could catalyse increases in the number of, and proportion of home deaths in the future. Scaling up these initiatives will help healthcare professionals better understand and support patients’ end-of-life preferences, if their wish was to spend their last days at home. Therefore, endeavoring to meet one’s preferences at the end-of-life cannot be constrained to just being the responsibility of the individual or family; it must encompass the resources and determined efforts of the community and society in creating a conducive, compassionate environment that supports people’s preferences of how and where they wish to die.
